# Spiperone enhances intracellular calcium level and inhibits the Wnt signaling pathway

**DOI:** 10.1186/1471-2210-9-13

**Published:** 2009-11-30

**Authors:** Desheng Lu, Dennis A Carson

**Affiliations:** 1 Moores Cancer Center, University of California San Diego (UCSD), La Jolla, CA 92093, USA

## Abstract

**Background:**

Wnt signaling affects fundamental development pathways by regulating cell proliferation and differentiation. Aberrant activation of Wnt/β-catenin signaling promotes the development of several cancers and is an attractive target for chemopreventive and chemotherapeutic agents.

**Results:**

In order to identify the novel antagonists for the Wnt/β-catenin pathway, we employed a cell-based Wnt reporter system (TOPflash) to screen a library of 960 known drugs. We identified spiperone, a psychotropic drug, as a novel Wnt inhibitor, which specifically blocks canonical Wnt signaling prior to the activation of β-catenin. The Wnt inhibitory function of spiperone is not associated with its dopamine-, serotonin- and sigma-receptor antagonist properties. Instead, spiperone increases intracellular calcium levels in a similar manner to thapsigargin, that also impedes Wnt signal transduction. Inhibition of protein kinase C had no effect on spiperone-mediated antagonism of Wnt signaling.

**Conclusion:**

Spiperone is a calcium regulator. It inhibits Wnt signaling by enhancing intracellular calcium levels.

## Background

The Wnt signaling pathway plays important roles in the regulation of cell proliferation, differentiation, and apoptosis [1-4]. In the canonical Wnt pathway, Wnt initiates signaling events by binding to a receptor complex, consisting of a member of the Frizzled (Fzd) family, and the low-density lipoprotein-receptor-related proteins (LRP) 5 or LRP6. Subsequently the cytoplasmic adaptor protein disheveled (Dvl) is phosphorylated and inhibits glycogen synthase kinase (GSK)-3β activity through its association with axin. Unphosphorylated β-catenin accumulates in the cytoplasm and translocates into the nucleus, where it interacts with members of T cell factor/lymphoid enhancer factor (TCF/LEF) family to activate transcription of Wnt target genes [1-4].

The β-catenin molecule is a key effector in the canonical Wnt pathway. However, not all Wnt proteins activate the β-catenin complex. Some Wnt family members, such as Wnt4, Wnt5a and Wnt11, are able to initiate β-catenin-independent Wnt signaling by binding to a Frizzled receptor and possibly the coreceptor, Knypek (Kny) or Ror1 or Ror2 [5,6]. This leads to release of intracellular calcium and the activation of enzymes such as calcium/calmodulin dependent protein kinase II (CamKII) and protein kinase C (PKC), which exert antagonistic activity on the canonical Wnt pathway. Calcium has been implicated as an important mediator of antagonism of canonical Wnt signaling, acting at multiple points in the canonical Wnt pathway [5,7,8].

Spiperone is a butyrophenone antipsychotic agent with dopamine and serotonin (5-HT) receptor antagonist properties [9-11]. It is also a high affinity ligand of sigma receptors [12]. Radiolabeled spiperone and its analogues have been widely used in assessing dopamine receptor function based on positron emission tomography (PET) in humans. In this study, we demonstrate that spiperone, but not other related psychotropic drugs blocks canonical Wnt signaling activated by Wnt and LRP6 by elevating intracellular calcium levels.

## Results

### Inhibition of canonical Wnt signaling by spiperone

**Figure 1:**
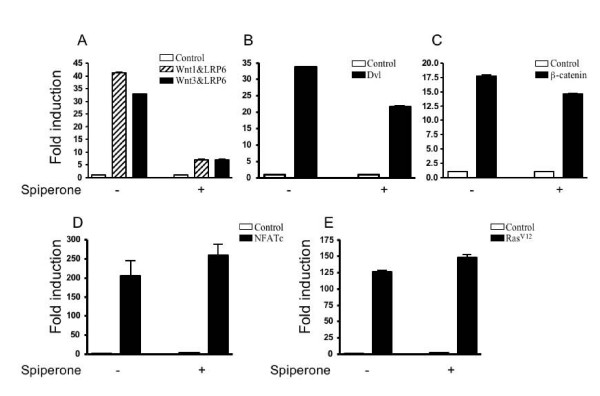
**Inhibition of Wnt signaling by spiperone**. HEK293 cells were cotransfected with a TOPflash reporter construct, along with vectors for: (A) control (pcDNA3 plasmid alone), Wnt1/LRP6, or Wnt3/LRP6; (B) control (pcDNA3 plasmid alone) or Dvl; (C) control (pcDNA3 plasmid alone) or β-catenin. (D) HEK293 cells were transfected with an NFAT-Luc reporter and an expression plasmid for NFATc. (E) HEK293 cells were transfected with an AP1-Luc reporter and an expression plasmid for H-Ras^V12^. After transfection for 24 h, the cells were treated with or without spiperone (5 μM) for another 24 h, and then harvested, and extracted for determination of luciferase activities. The β-galactosidase control plasmid was used to correct for transfection efficiency. The results are expressed as fold induction of luciferase activity normalized to a β-galactosidase control, and are the means of three experiments ± SEM.

To identify antagonists of canonical Wnt signaling, we used a cell-based TOPflash reporter system to screen the Gen-plus drug library (Microsource) that contains 960 FDA-approved drugs. In this system, transfected Dvl (an upstream activator of the Wnt pathway) stimulated TCF/LEF response elements in the TOPflash reporter gene. Inhibitors of Wnt signaling were identified based on their ability to block the transcription of the reporter gene, but not a control gene. Small molecular compounds were screened at 10 μM and 50 μM. The initial screen identified spiperone as an antagonist of Wnt signaling. To confirm the Wnt inhibitory effect of spiperone, the TOPflash reporter was activated by Wnt1/LRP6 or Wnt3/LRP6, Dvl and β-catenin, respectively, in transient transfection assays. Treatment with 5 μM spiperone only weakly inhibited Dvl- or β-catenin-mediated signaling (Figure. 1B&1C), whereas a more potent effect was observed at higher concentration (≥ 10 M) (data not shown). Surprisingly, treatment of the same dose of spiperone strongly blocked Wnt signaling activated by Wnt1/LRP6 and Wnt3/LRP6, respectively (Figure 1A). In control experiments, spiperone did not inhibit signals from reporter genes for NFAT and activator protein 1 (AP-1) (Figure 1D &1E). These results suggest that spiperone may specifically inhibit Wnt signaling by targeting the Wnt/LRP complex. Spiperone was chosen for the further study because of its highly selective inhibitory effect on Wnt/LRP-mediated signaling.

### Antipsychotic spiperone analogs have no inhibitory effect on Wnt signaling

To determine whether spiperone-mediated inhibition of Wnt signaling is associated with its antipsychotic effect, we examined other psychotropic drugs for their effects on Wnt signaling. These ligands include serotonin 5-HT1 receptor antagonists (pindolol, UH-301, WAY100635 and NAN-190), 5-HT1 receptor agonist (8-OH-DPAT), 5-HT2 receptor antagonists (ketaserin and SB-204741), additional serotonin receptor antagonists (clozapine and SB-269970) and a sigma receptor ligand (DTG) [13-15]. NAN-190 and clozapine have been shown to be dopamine receptor antagonists, whereas UH-301 and WAY100635 are dopamine receptor agonists [16,17].

**Figure 2:**
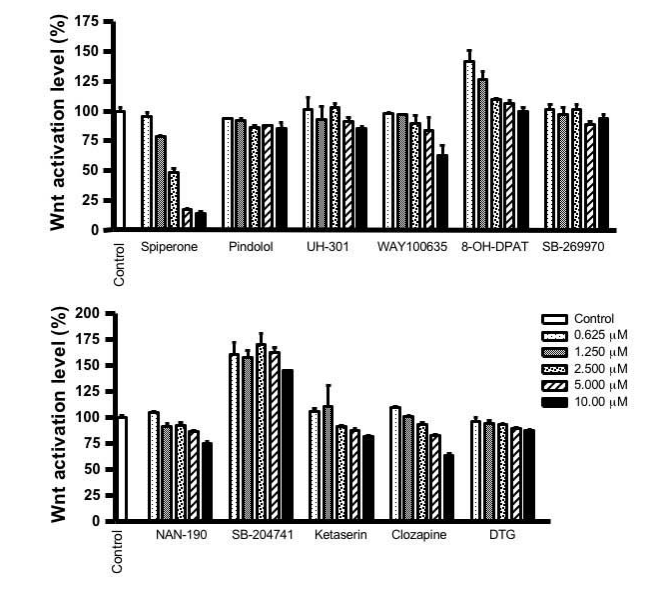
**Effect of psychotropic drugs on Wnt signaling activated by Wnt1 and LRP6**. The TOPflash reporter was transfected into HEK293 cells with expression plasmids encoding Wnt1 and LRP6. After transfection, cells were treated with increasing concentrations of antipsychotic spiperone analogs for 24 h, as indicated. Then cells were harvested and luciferase activities were determined.

The TOPflash reporter was transfected into HEK293 cells with expression plasmids for Wnt1 and LRP6. The cells were treated with increasing concentrations of the various psychotropic drugs. Surprisingly, none of the drugs tested except spiperone had the ability to block Wnt1/LRP6-mediated signaling (Figure. 2), suggesting that inhibition of Wnt signaling is not related to compound interactions with dopamine, serotonin or sigma receptors.

### Spiperone displays a similar Wnt inhibition profile to that of ionomycin and thapsigargin

**Figure 3:**
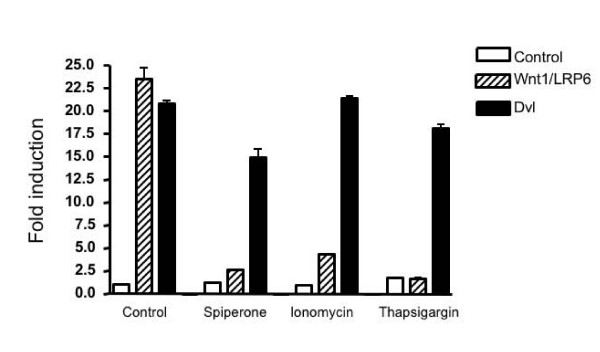
**Spiperone, ionomycin and thapsigargin inhibit Wnt1- and LRP6-initiated Wnt signaling**. HEK293 cells were transfected with a TOPflash reporter and expression plasmids for Wnt1, LRP6 and Dvl as indicated in the figure. After transfection for 24 h, cells were grown with 5 μM spiperone, 2 μM ionomycin, 50 nM thapsigargin and vehicle control for another 24 h. Then luciferase values were determined.

A previous study demonstrated that ionomycin strongly inhibited Wnt pathway activation induced by a Wnt ligand, but had much less effect on β-catenin-mediated Wnt signaling [7]. The inhibition profile exerted by ionomycin treatment is similar to that of spiperone. Accordingly, we examined the Wnt inhibitory effects of spiperone, ionomycin and thapsigargin. Ionomycin and thapsigargin are well known agents that mobilize intracellular calcium. As shown in Figure 3, all three agents dramatically inhibited Wnt signaling activated by Wnt1 and LRP6. In contrast, spiperone and thapsigargin weakly inhibited Dvl-mediated signaling, whereas ionomycin had almost no inhibitory effect at the concentration tested (Figure. 3). We observed some inhibition in Dvl-mediated transcription at higher drug concentrations, associated with cell toxicity (data not shown). These results suggest that spiperone, ionomycin and thapsigargin inhibit the Wnt signaling pathway through a similar mechanism, that involves changes in intracellular calcium levels.

### Spiperone effects on calcium mobilization

**Figure 4:**
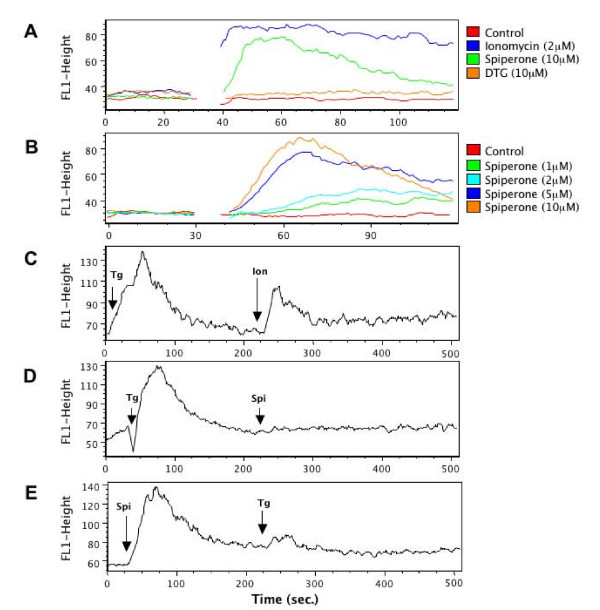
**Spiperone induce a rise in intracellular calcium levels**. (A) Tracing of fluo-4 fluorescence in cells stimulated with 10 μM spiperone, 10 μM DTG and 2 μM ionomycin over time. Drugs were added at 30 sec. (B) Tracing of fluo-4 fluorescence in cells stimulated with increasing doses of spiperone. Drugs were added at 30 sec. (C) Pretreatment with 1 μM thapsigargin could not abolish ionomycin-induced calcium release. Drugs were added at 10 and 225 sec as indicated by the arrowheads. (D) Thapsigargin blocks the subsequent cellular calcium responses to spiperone. Intracellular Ca^2+ ^was recorded after stimulation with 1 μM thapsigargin and 10 μM spiperone. Drugs were added at 30 and 225 sec as indicated by the arrowheads. (E) Spiperone prevented thapsigargin-induced calcium increase. Intracellular calcium was traced after stimulation with 10 μM spiperone and 1 μM thapsigargin. Drugs were added at 30 and 225 sec as indicated by the arrowheads.

In order to assess whether spiperone has the ability to elevate intracellular calcium levels, Fluo-4 FACS analysis was used. As shown in Figure 4A, spiperone and ionomycin increased intracellular calcium levels, whereas DTG had no affect under similar conditions. In cells treated with ionomycin, calcium levels remained elevated throughout the recording period (Figure. 4A). In contrast, calcium signals induced by spiperone were relatively transient, suggesting that the two drugs alter calcium concentrations by different mechanisms. The effect of spiperone was dose-dependent, with a maximal increase in calcium levels occurring at 5 μM (Figure. 4B).

Ionomycin, a calcium ionophore, increases calcium flux across membranes by shielding its polar electrical charge from the apolar lipid bilayer [18]. Thapsigargin is an inhibitor of the calcium ATPase pump, which causes calcium release from the endoplasmic reticulum into the cytoplasm, leading to perturbations in calcium homeostasis [19]. As expected, pretreatment with 1 μM thapsigargin could not abolish ionomycin-induced calcium release (Figure 4C). However, thapsigargin blocked the subsequent cellular calcium response to spiperone. (Figure 4D). Similarly, pretreatment with spiperone prevented the thapsigargin-induced calcium increase (Figure 4E). These results suggest that spiperone and thapsigargin may act by a similar mechanism to enhance intracellular calcium levels.

### The PKC inhibitor GF109203X does not affect spiperone-mediated inhibition of Wnt signaling

**Figure 5:**
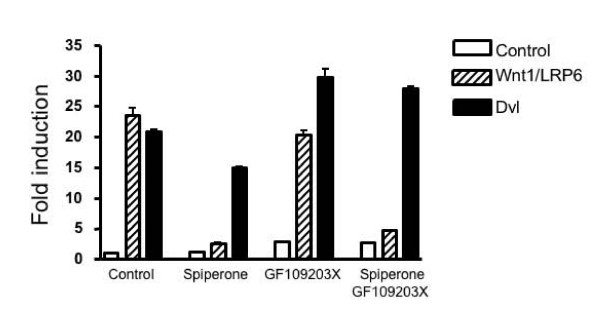
**The protein kinase C inhibitor GF109203X does not affect spiperone-mediated inhibition of Wnt signaling**. A TOPflash reporter was transfected into HEK293 cells with expression plasmids for Wnt1, LRP6 and Dvl as indicated in figure. After transfection, cells were treated with 5 μM spiperone, 5 μM GF109203X and combined use of two drugs for another 24 h. Then luciferase values were determined.

Calcium is a ubiquitous second messenger used to regulate a wide range of cellular processes. When intracellular calcium is increased, it interacts with protein kinase C (PKC), calmodulin, and with other intracellular molecules, to activate downstream signaling pathways. To explore the role of PKC in spiperone-mediated inhibition of Wnt signaling, a PKC inhibitor GF109203X was tested. As indicated in Figure 5, GF109203X had no effect on spiperone-mediated inhibition of Wnt signal transduction induced by Wnt1 and LRP6, while GF109203X alone slightly enhanced the Dvl-initiated signaling pathway (Figure 5).

## Discussion

Previous studies have demonstrated that noncanonical Wnt family members repress canonical Wnt signaling by inducing a calcium influx [5,7,20-22]. Some regulators of calcium signaling also have been shown to inhibit canonical Wnt signaling [23,24]. Maye et al. reported that an increase in intracellular calcium concentration induced by ionomycin led to inhibition of the canonical Wnt pathway [7]. They also noted that ionomycin strongly blocked Wnt1-activated signaling, whereas its inhibition became much less effective with downstream pathway activators such as Dvl and β-catenin [7]. In this study, we identified spiperone as a calcium regulator. Like ionomycin and thapsigargin, spiperone specifically targets the Wnt/LRP6 complex, and increases intracellular calcium levels. However, it is unclear how calcium blocks Wnt-activated signaling. Our results indicate that blocking PKC activity by GF109203X does not alter spiperone-mediated inhibition of Wnt signaling, suggesting that the PKC activation pathway is not involved.

A recent study showed that ionomycin is a negative regulator of β-catenin/TCF signaling in colon cancer cells and its inhibitory mechanism is related to the decreased nuclear β-catenin products and to the suppressed binding of TCF complexes to consensus DNA [8]. However, this study did not check the effect of ionomycin on Wnt signaling activated by Wnt and LRP coreceptor. In our experiment, we showed that spiperone and thapsigargin weakly inhibited Dvl-mediated signaling (Figure 3). We also noted that ionomycin elicited some inhibitory effect on Dvl- and β-catenin-mediated signaling at concentrations equal to and above 5 μM (data not shown). Therefore, in addition to its inhibition function on Wnt/LRP complex, ionomycin may target multiple steps of the canonical Wnt pathway.

Spiperone is a psychotropic agent that acts as a potent dopamine D_2_, serotonin 5-HT1A, and serotonin 5-HT2A antagonist [9-11], and binds to sigma receptors with high affinity [12]. Spiperone also has been reported to have immunosuppressive effects in the mouse, and this action may be independent of its serotonin or dopamine receptor blocking properties [25]. Among the several related psychotropic drugs analyzed in the present study, only spiperone significantly blocked Wnt signaling. It will be interesting to determine whether spiperone-mediated Wnt inhibition is associated with its immunosuppressive function, insofar as Wnt-signaling is required for lymphocyte development.

During the preparation of this manuscript, Liang et al. [26] reported that spiperone stimulated calcium-dependent chloride secretion through a protein tyrosine kinase-coupled phospholipase C-dependent pathway, which supports our findings.

## Conclusion

In summary, our result clearly demonstrated that spiperone is a calcium regulator. It specifically blocks canonical Wnt signaling by elevating intracellular calcium levels. This drug may have chemopreventive or chemotherapeutic utility in malignancies associated with abnormal Wnt activation.

## Methods

### Chemical reagents

Spiperone, ionomycin, thapsigargin (Tg), pindolol, UH-301, WAY100635, 8-OH-DPAT, SB-269970, NAN-190, SB-204741, Ketaserin, Clozapine, DTG (1,3-di-o-tolylguanidine) and GF109203X were purchased from Sigma-Aldrich (St. Louis, MO). A Gen-plus collection of 960 known drugs was obtained from Microsource (Gaylordsville, CT).

### Transfection and screening of drug library

The human embryonic kidney cell line HEK293 (American Type Culture Collection, Rockville, MD) was transfected using the FuGene transfection reagent (Roche Diagnostics GmbH, Mannheim, Germany) according to the manufacturer's instruction.

The reporter plasmid TOPflash was a gift from H. Clevers (University of Utrecht, Utrecht, The Netherlands). The NFAT-Luc and AP1-Luc reporters were purchased from BD Biosciences. The expression plasmids encoding Wnt1, Wnt3, LRP6, Dvl, β-catenin, NFATc and H-ras^V12 ^have been described previously [27,28].

For screening of the drug library, HEK293 cells were grown for at least 24 h in 10 cm plates prior to transfection. At ~50% confluence, cells were transfected with 5 μg of TOPflash reporter, 1 μg expression vector for Dvl, 1 μg of control plasmid pCMXβgal and carrier DNA pcDNA3 plasmid for a total of 10 μg/plate. After transfection for 24 h, cells were harvested and dispersed in 96-well microtiter plates. Then the cells were treated with the different agents, generally at 10 μM and 50 μM for the initial screen. After overnight incubation, the cells were lysed in 1× potassium phosphate buffer, pH 7.8, containing 1% Triton X-100, and luciferase activities were assayed in the presence of substrate using a microtiter plate luminometer (MicroBeta TriLux, Gaithersburg, MD). The luciferase values were normalized for variations in transfection efficiency using the β-galactosidase internal control. Spiperone, and other compounds that were scored positive, had ≥30% inhibition of TOPflash activity when compared to the designated control cultures. In other experiments, transient transfections were performed in 12-well plates. HEK293 cells were transfected with 0.5 μg of reporter plasmid, 0.1 μg of control plasmid pCMXβgal, 0.1-0.2 μg expression plasmids, and carrier DNA pcDNA3 plasmid for a total of 1 μg/well. After 16 h, the cells were washed and treated with 5 μM spiperone or solvent (DMSO) for 24 h. Then luciferase values were determined. In the Results section, data are expressed as fold stimulation of luciferase activity compared to the basal level. All the transfection results represent means of a minimum of three independent transfections assayed in duplicate, ± the standard error of the mean (SEM).

### Calcium measurements

Changes in intracellular Ca^2+ ^concentration were measured with the calcium sensitive dye Fluo-4 (Fluo-4/AM, Molecular Probes). Briefly, HEK293 cells (2 × 10^6 ^cells/ml) were incubated with 2 μM Fluo-4/AM at 37°C for 30 min in Hanks balanced salt solution (HBSS) containing 5 mM KCl, 0.4 mM KH_2_PO_4_, 0.8 mM MgSO_4_, 137 mM NaCl, 0.3 mM Na_2_HPO_4_, 5.5 mM glucose, 1.26 mM CaCl_2_, 0.5 mM MgCl_2_. Cells were washed 2 times with HBSS and then suspended in 2 ml HBSS. Samples were analyzed for 30 sec to establish a base line and then stimulated with 2 μM ionomycin, 10 μM DTG, 1 μM thapsigargin and different concentrations of spiperone as indicated in figure 4. The intracellular calcium concentration was measured by flow cytometry using a FACSCalibur (Becton Dickinson). The calcium concentration was expressed as Fluo-4/AM fluorescence intensity using the FL1 channel. At least 10,000 cells were counted to evaluate the intracellular calcium elevation.

## Competing interests

The authors declare that they have no competing interests.

## Authors' contributions

DL participated in the design of the study, carried out the experiments and drafted the manuscript. DAC participated in the design of the study and drafted the manuscript. All authors read and approved the final manuscript.
